# Hierarchical Multi-Scale Convolutional Neural Networks for Hyperspectral Image Classification

**DOI:** 10.3390/s19071714

**Published:** 2019-04-10

**Authors:** Simin Li, Xueyu Zhu, Jie Bao

**Affiliations:** 1 Department of Electronic Engineering, Tsinghua University, Beijing 100084, China; lism14@mails.tsinghua.edu.cn; 2 Department of Mathematics, University of Iowa, Iowa City, IA 52242, USA; xueyu-zhu@uiowa.edu

**Keywords:** hyperspectral image (HSI) classification, convolutional neural networks (CNNs), bidirectional LSTM, multi-scale features

## Abstract

Deep learning models combining spectral and spatial features have been proven to be effective for hyperspectral image (HSI) classification. However, most spatial feature integration methods only consider a single input spatial scale regardless of various shapes and sizes of objects over the image plane, leading to missing scale-dependent information. In this paper, we propose a hierarchical multi-scale convolutional neural networks (CNNs) with auxiliary classifiers (HMCNN-AC) to learn hierarchical multi-scale spectral–spatial features for HSI classification. First, to better exploit the spatial information, multi-scale image patches for each pixel are generated at different spatial scales. These multi-scale patches are all centered at the same central spectrum but with shrunken spatial scales. Then, we apply multi-scale CNNs to extract spectral–spatial features from each scale patch. The obtained multi-scale convolutional features are considered as structured sequential data with spectral–spatial dependency, and a bidirectional LSTM is proposed to capture the correlation and extract a hierarchical representation for each pixel. To better train the whole network, weighted auxiliary classifiers are employed for the multi-scale CNNs and optimized together with the main loss function. Experimental results on three public HSI datasets demonstrate the superiority of our proposed framework over some state-of-the-art methods.

## 1. Introduction

Hyperspectral images (HSIs) [1] often contain hundreds of spectral bands varying from visible wavelength to short infrared light. This rich information enables us to distinguish different materials which look similar to the naked eye or the conventional RGB cameras. To benefit from this type of data, HSI classification has become one of the most important tasks for various applications [2,3,4,5,6] and many classification methods have been proposed.

In the last decades, many pattern recognition approaches, such as decision tree [7], support vector machine (SVM) [8], random forest [9], and maximum likelihood estimate (MLE) [10] have been utilized to perform HSI classification. Among these traditional methods, SVM is considered as a stable and efficient method for HSI classification since it can handle the “curse of dimensionality” problem [11] and requires a relatively small size of training data. In recent years, contextual information [12] has been proven to be effective for HSI classification, leading to a growing interest in the research of joint spectral–spatial features. Earlier methods include Gabor filters [13], gray-level co-occurrence matrices [14] and mathematical morphological profiles [15]. Though these methods could improve the classification results to some extent, the handcrafted features are strongly dependent on the prior information assumed by the practical practitioners. Besides, the obtained joint spectral–spatial features are relatively shallow and have a poor adaption to the spatial environment changes.

More recently, deep neural networks have attracted much attention due to their excellent performances in computer vision tasks [16,17,18]. Motivated by these successful applications, deep learning models have also been introduced to remote sensing field. At the early stage, deep belief networks (DBNs) [19] and stacked autoencoders (SAEs) [20] were first applied in HSI classification by Chen. The whole training process contains two phases, namely unsupervised pre-training and supervised classification. During the unsupervised pre-training process, the parameters of the whole network have been initialized to a relatively optimized stage, then they are fine-tuned given the labels through a supervised logistic regression classifier. Later, convolutional neural networks (CNNs) were introduced to HSI classification and have been widely applied due to its ability of hierarchically extracting deeper features with much fewer parameters. Hu et al. [21] constructed a 1D CNN containing convolutional layers, pooling layers and fully connected layers to extract deep spectral features for each pixel. Later, Makantasis et al. [22] applied CNNs to spatial dimension to include spatial features and obtained a joint spectral–spatial representation. Moreover, to reduce the computation complexity, principal component analysis (PCA) has been applied to HSI cube first for dimension reduction. In [23], Chen et al. proposed a 3D-CNN model where the convolutional operation is conducted simultaneously on both spectral and spatial dimensions.

As an important branch of deep neural networks, recurrent neural networks (RNNs) [24] have gained significant attention for their unique remembering ability and excellent performance in dealing with structured sequential data in recent years. Since the spectral dimension of HSI has strong correlation and the temporal variability of a sequential signal is similar to the spectral variability of a hyperspectral pixel [25], RNNs have also been introduced to HSI classification field. In [25], the spectrum of each hyperspectral pixel is treated as a sequential data for the first time and the authors utilized a modified gated recurrent unit (GRU) network to model the spectral dependency and produce the classification results. In [26], Zhou et al. applied a long-short-term-memory (LSTM) network to extract spectral features and spatial features respectively, and obtained joint spectral–spatial classification results.

Although these DL-based methods have made substantial improvements, seldom of them ever consider the scale variation problems in remote sensing images (e.g., roofs with different shapes and sizes), leading to the strong dependency on the empirical parameters (e.g., window size). Since the objects in HSI plane have various sizes and shapes, conventional methods with the single input scale as mentioned above might fail to capture crucial scale-dependent features [27] over the image plane. Therefore, it is difficult to assign appropriate parameters to generate spatial features for all kinds of objects. To address this problem, multi-scale contextual and structural information in the spatial domain requires to be considered. In [28], Zhao proposed multi-scale convolutional neural networks (MCNNs) which utilizes the first three principal components (PCs) of HSI to generate pyramid image samples at each PC respectively and trained these image samples for spatial features. Then he concatenated the obtained spatial features with spectral information and applied majority voting for the final classification. Although the scale features were obtained in this method, the inherent spatial dependency among different spatial scales are failed to discuss.

Motivated by this, we proposed a novel deep learning framework, namely hierarchical multi-scale CNNs with auxiliary classifiers (HMCNN-AC) for HSI classification in this paper. As mentioned above, the single input spatial scale would limit the observation field and fail to capture the scale-dependent information. Hence, a multi-scale feature extraction technique is introduced to address this problem. First, multi-scale hyperspectral patches for each pixel are generated with various spatial scales. Then we apply multi-scale CNNs to these input patches to extract spectral–spatial features at different input scales simultaneously. Since these multi-scale patches are spatial-dependent and share the same central spectrum with the same class label, the extracted multi-scale features are considered as sequential structured data with spatial–spectral correlation. Thus, a bidirectional LSTM is adopted to characterize the sequential property and extract a hierarchical representation for each hyperspectral pixel. To better train the whole network, auxiliary classifiers are added as constraints for the multi-scale CNNs and optimized together with the main loss during the training process. The main contributions of this paper are summarized as follow:

1. An end-to-end framework (HMCNN-AC) combining multi-scale CNNs and a bidirectional LSTM is proposed to generate discriminative features with spectral information, spatial information and scale-dependency for HSI classification.
2. To address the limitation of the single input spatial scale and effectively utilize the spatial contexture, a multi-scale CNN based technique is introduced to extract spectral–spatial features at various input scales simultaneously. In this way, different shapes and sizes of objects in HSI could be considered during classification and the multi-scale features will make the performance more robust compared with the single scale input.
3. A bidirectional LSTM is proposed to capture the dependency and correlation between the obtained multi-scale features from a sequential perspective and output a hierarchical representation for each pixel. Compared with the conventional concatenation method to simply concatenate multi-scale features together, we can make full use of the correlation between multi-scale features without losing scale-dependent information by adopting the bidirectional LSTM.
4. In order to better train the whole network, weighted auxiliary classifiers are employed for multi-scale convolutional features and optimized together with the main loss during the training process. This helps to fully optimize the parameters in multi-scale CNNs and obtain robust convolutional features without adding too many extra parameters.

The rest of the paper is organized as follows. Section 2 reviews the classical deep learning models used in this paper, namely CNNs and LSTM networks. In Section 3, we give a detailed description of our proposed HMCNN-AC framework. Experimental analysis and a comparative evaluation of other baseline methods are reported in Section 4. Section 5 presents the final conclusion and gives directions to the future work.

## 2. Background of CNNs and LSTM

### 2.1. CNNs

Convolutional neural networks (CNNs) have attracted much attention due to their outstanding performance in computer vision tasks [29,30]. Typical CNNs consist of convolutional layers, pooling layers, and a fully connected layer. The convolutional layers are composed of multiple convolutional kernels. Each kernel is a weight matrix and generates a distinct feature map via the convolutional operation with the input, which can be formulated as:

(1) $$\begin{array}{c} x_{i}^{j}=F\left(W_{i}^{j}*x_{i-1}+b_{i}^{j}\right), \end{array}$$

where $x_{i}^{j}$ is the *j*th channel’s feature map of the present layer *i*, $W_{i}^{j}$ is the *j*th convolutional kernel matrix at layer *i*, $b_{i}^{j}$ is the bias term, $x_{i-1}$ is the output feature maps of the (i−1)th convolutional layer, the symbol ∗ represents convolutional operation, and F(·) is an activation function.

Pooling layers are often after the convolutional layers to further reduce the redundancy by partitioning the input data into a set of non-overlapped sub-regions and returning the average or maximum values locally. Both convolutional layers and pooling layers can be repeated multiple times to obtain the representative features. Finally, a fully connected layer is followed to further process the extracted features and a multinomial logistic regression (MLR) layer is added at the end to convert the output convolutional features into category or regression results.

### 2.2. LSTM and Bidirectional LSTM

Recurrent neural networks (RNNs) are an important class of deep neural networks which has “memory” functionality and can remember past state information while dealing with the current state. This powerful function makes RNNs extremely suitable for processing sequential inputs. However, the traditional RNNs are difficult to train in practice due to the vanishing or exploding gradient [31].

**Figure 1:**
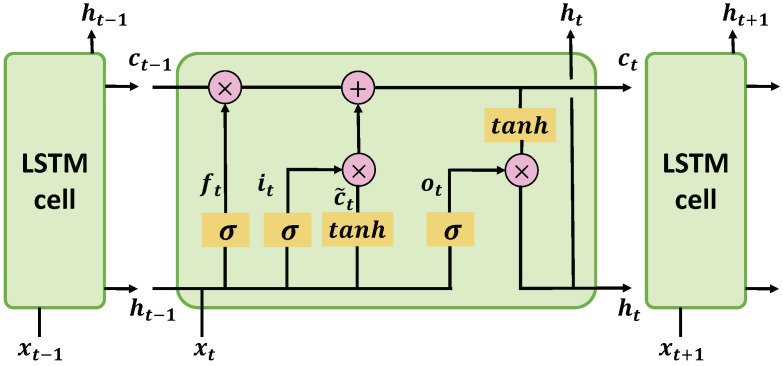
A basic cell of LSTM. By explicitly introducing a memory cell *c_t_* together with some control gates *f_t_*, *i_t_* and *o_t_* to decide the information flow, LSTM can capture long-term dependencies of the sequential inputs.

To address this issue, the long short-term memory (LSTM) network [32] was developed to capture the long-term dependencies by designing a more sophisticated recurrent unit. The diagram of a basic LSTM building block is presented as Figure 1. Compared with the traditional RNNs, LSTM explicitly introduces a memory cell into the network together with some control gates to decide the information flow. As we can see, every unit of the network takes three inputs, namely the current input data $x_{t}$, the previous hidden state $h_{t-1}$ and the previous memory cell $c_{t-1}$. Then we use them to calculate the following values. The current hidden state $h_{t}$ is obtained by:

(2) $$\begin{array}{c} h_{t}=o_{t}tanh\left(c_{t}\right), \end{array}$$

where tanh(·) is the hyperbolic tangent function [33] and $o_{t}$ is the output gate at current state which determines the percentage of the exposed memory content. The $o_{t}$ is determined as:

(3) $$\begin{array}{c} o_{t}=\sigma \left(W_{oi}x_{t}+W_{oh}x_{t}+b_{o}\right), \end{array}$$

where the σ(·) is the sigmoid function, $W_{oi}$ and $W_{oh}$ represent the weight matrices of the input-output and the hidden-output respectively, and $b_{o}$ is the bias of this layer. The $c_{t}$ is the memory cell of the current state and it is updated by adding some new content of the candidate memory cell ${\tilde{c}}_{t}$ and discarding part of the previous memory cell $c_{t-1}$, as described in Equation (4):

(4) $$\begin{array}{c} c_{t}=i_{t}⊙{\tilde{c}}_{t}+f_{t}⊙c_{t-1}, \end{array}$$

where the input gate $i_{t}$ controls the extent to which the candidate memory cell ${\tilde{c}}_{t}$ will be added to the current memory cell $c_{t}$, and the forget gate $f_{t}$ modulates the percentage of the previous memory cell $c_{t-1}$ should be forgotten. The operation ⊙ is an element-wise multiplication and the new candidate memory cell ${\tilde{c}}_{t}$ is obtained by:

(5) $$\begin{array}{c} {\tilde{c}}_{t}=tanh\left(W_{ci}x_{t}+W_{ch}h_{t-1}+b_{c}\right). \end{array}$$

The input gate $i_{t}$ and forget gate $f_{t}$ are calculated as follows:

(6) $$\begin{array}{c} i_{t}=\sigma \left(W_{ii}x_{t}+W_{ih}h_{t-1}+b_{i}\right), \end{array}$$

(7) $$\begin{array}{c} f_{t}=\sigma \left(W_{fi}x_{t}+W_{fh}h_{t-1}+b_{f}\right). \end{array}$$

The LSTM explores the dependencies of a current state with the previous states. However, when it comes to the non-time sequential vectors, the dependencies exist in both the forward direction and the backward direction. To solve this problem, a bi-directional LSTM [34] was proposed to utilize both forward and backward relationship of a sequential data. In that case, we first apply a forward LSTM to the input sequence to obtain a forward output, and then a backward LSTM is applied to obtain a backward output. The final output of this layer is a concatenated version of the forward vector and the backward vector.

## 3. Proposed Methods

**Figure 2:**
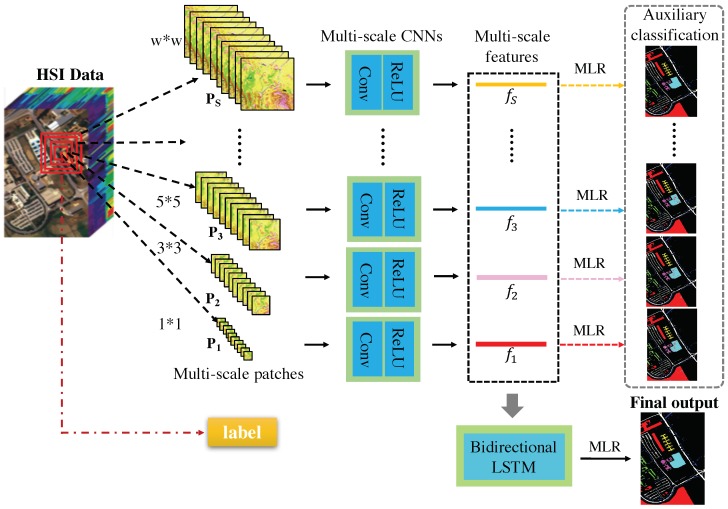
The architecture of the proposed HMCNN-AC consisting of multi-scale CNNs and a bidirectional LSTM. For each pixel in HSI, the generated multi-scale patches are sent to the multi-scale CNNs with auxiliary classifiers to extract multi-scale spectral–spatial features, then a bidirectional LSTM is employed to explore the scale-dependency of multi-scale features and output a hierarchical representation for the final supervised classification.

In this section, we give a detailed description of our proposed hierarchical multi-scale CNNs with auxiliary classifiers (HMCNN-AC) for HSI classification. The whole framework is shown in Figure 2. To solve the limitation of the single input scale in conventional methods, multi-scale CNNs are employed to extract multi-scale convolutional features at different spatial scales. In addition, to fully explore the dependency and correlation of the obtained multi-scale features, a bidirectional LSTM is adopted to characterize the sequential property and extract a hierarchical representation for each hyperspectral pixel. Moreover, to better train the whole network, weighted auxiliary classifiers are employed for these multi-scale convolutional features. The whole framework is trained in an end-to-end supervised manner and all the parameters are optimized by mini-batch stochastic gradient descent (SGD) algorithm [35].

### 3.1. Multi-Scale Convolutional Feature Extraction

Traditional CNNs can only extract deep spectral–spatial features from receptive fields with one single input scale. Since objects in remote sensing images often appear with various shapes and sizes, the fixed input scale will limit the effectiveness of spatial feature integration and fail to capture the scale-dependent information. In order to accommodate the objects with different scales and effectively utilize the spatial contexture, multi-scale CNN based technique is introduced to our proposed framework. Specifically, our multi-scale convolutional feature extraction is composed of two main phases: multi-scale patches generation and convolutional feature extraction.

To construct multi-scale patches, a sub-region image set with *S* scales ${\left\{P_{s}\right\}}_{s=1}^{S}$ is generated for each pixel from the original HSI cube. The first scale patch $P_{1}$ is just the central spectrum itself with the size of 1×1×D, where *D* represents the number of spectral bands. The second scale patch $P_{2}$ contains $P_{1}$ but with a larger spatial scale of 3×3×D. In that case, an upper scale patch always contains the lower scale patches, which results in a spatial dependency among multi-scale patches. Our last scale patch $P_{s}$ contains all the lower patches with a size of w×w×D, where *w* is the largest input scale, namely the original input window. The window size *w* is carefully selected so that it would include enough spatial information without increasing the outliers’ disturbance and the computing complexity. For each pixel, all these generated multi-scale patches are spatial-dependent and share the same central spectrum with the same class label, as seen in Figure 2. By utilizing multi-scale patches, we would not only take multi-scale spatial information into account, but also put more emphasis on the central spectra and reduce the unwanted noise.

In our second phase, we perform convolutional operations on the generated multi-scale patches to extract spectral–spatial features at different input scales. To preserve as much spatial information as possible, we exclude pooling layers from the multi-scale CNNs and treat each scale patch as a conventional image with multiple channels on its own [23,36,37]. The convolutional operator is formulated as below:

(8) $$\begin{array}{c} x_{i}^{j}=F\left(W_{i}^{j}*x_{i-1}+b_{i}^{j}\right), \end{array}$$

where $x_{i}^{j}$ denotes the *j*th feature map at the *i*th layer; $x_{i-1}$ is the output of the (i−1)th convolutional layer; F(·) stands for the activation function; $b_{i}^{j}$ refers to the bias term; and $W_{i}^{j}$ is the *j*th convolutional kernel at the layer *i* with the depth equaling to the channel number of $x_{i-1}$. For the first convolutional layer, the depth of $W_{1}^{j}$ equals to the number of spectral bands *D*. The detailed architecture description of the multi-scale CNNs is listed in Table 1. For the proposed model, we adopt the ReLU [38] function as the activation function which is defined as follow:

(9) $$\begin{array}{c} F(x)=max(0,x). \end{array}$$

After the convolutional layers, we adopt a fully connected layer for each spatial scale to obtain the multi-scale convolutional features $f_{1},f_{2},\ldots ,f_{S}$ for each pixel. Since these multi-scale features contain discriminative characteristics from both spectral dimension as well as their respective spatial scales, we can use them to improve the HSI classification results.

### 3.2. Hierarchical Feature Learning

A conventional way to process the obtained multi-scale convolutional features is to concatenate them together into a 1-D vector directly. However, this simple concatenation-based method ignores the inherent correlation of the multi-scale features and will lead to the loss of scale-dependent information. Since the multi-scale patches for each pixel are spatial-dependent and share the same central spectrum with the same class label, we consider the extracted multi-scale features as sequential structured data with spatial–spectral dependency. Thus, a bidirectional LSTM is proposed to characterize the sequential property and extract a hierarchical representation for each hyperspectral pixel.

We apply one or two layers of bidirectional LSTM to the obtained multi-scale features $f_{conv}$, depending on the performance of different HSI datasets:

(10) $$\begin{array}{c} f=BiLSTM(f_{conv}), \end{array}$$

where $f_{conv}=\left[f_{1},f_{2},\ldots ,f_{S}\right]$ is the multi-scale convolutional feature sequence with length *S*, *S* is the scale number of generated multi-scale patches for each pixel; and BiLSTM is the bidirectional LSTM operation which is introduced in Section 2. The obtained hierarchical feature *f* is regarded as the most representative feature for each pixel and will be used for the final HSI classification.

### 3.3. Label Prediction

After the multi-scale CNNs and the bidirectional LSTM networks, we feed the output feature *f* to a MLR layer for the final classification. The output size of the MLR is the same as the total number of HSI classes and we use the Softmax function [39] as the activation function. The label of each pixel is determined by the class with the largest probability:

(11) $$\begin{array}{c} y=\Phi (W_{MLR}f+b_{MLR}), \end{array}$$

where $W_{MLR}$ and $b_{MLR}$ are the weight matrix and bias of the MLR layer, *y* is the predicted label and Φ(·) is the MLR function which is defined as:

(12) $$\begin{array}{c} \Phi \left(x\right)=\underset{i}{\mathrm{argmax}}\left(\frac{e^{x_{i}}}{\sum_{j}e^{x_{j}}}\right). \end{array}$$

### 3.4. Weighted Auxiliary Classifiers

A main difference between our proposed method and the traditional deep learning-based HSI classification methods is the adoption of the weighted auxiliary classifiers for our multi-scale CNNs. The motivation of employing these auxiliary classifiers is to better train the sub-networks of the multi-scale CNNs in our proposed architecture and acquire robust multi-scale convolutional features, especially for a deep model consisting of two neural networks. Since the final output feature *f* is closer to the bidirectional LSTM part, the parameters in the bidirectional LSTM might have a dominant position during the training process, while the parameters in the multi-scale CNNs may not get fully optimized.

To address this issue, auxiliary classifiers are added as constraints for the multi-scale convolutional features to perform sub-classification. The predicted label for each multi-scale feature is given as:

(13) $$\begin{array}{c} y_{i}=\Phi \left(W_{aux}^{i}f_{i}+b_{aux}^{i}\right), \end{array}$$

where $f_{i}$ is the *i*th scale convolutional feature from the multi-scale CNNs and i=1,2,…,S, $W_{aux}^{i}$ and $b_{aux}^{i}$ are the weight matrix and bias of *i*th auxiliary classifier, Φ(·) is the MLR function which produces the *i*th auxiliary predicted label $y_{i}$. The total loss *L* of our proposed HMCNN-AC contains one main loss $L_{main}$ as well as *S* weighted auxiliary losses $L_{aux}^{i}$, and measures the total difference between the predicted outputs and the real labels, which is defined as:

(14) $$\begin{array}{c} L=L_{main}+\sum_{i=1}^{S}\alpha_{i}L_{aux}^{i}, \end{array}$$

where $L_{main}$ is the main loss of the whole framework, $L_{aux}^{i}$ is the *i*th auxiliary loss for the *i*-th scale convolutional feature $f_{i}$; $\alpha_{i}$ is the weight for its corresponding auxiliary loss and will be discussed in later experiments; *S* is the number of multi-scale features. The whole network is trained in an end-to-end manner and all the parameters are optimized simultaneously by minimizing the total loss function *L*.

## 4. Experimental Results and Analysis

In order to evaluate our proposed model, three publicly available HSI datasets [40] are utilized to perform classification. We choose several other HSI classification methods, including SVM, extended morphological profiles with SVM (EMP-SVM) [15], stacked autoencoder (SAE-PCA) [20], CNN-based structure on spatial dimension (CNN-MLR) [22], LSTM [25] and MCNN [28], as baselines for a comparative evaluation. To evaluate the effectiveness of the adopted auxiliary classifiers and the bidirectional LSTM in our proposed method, we also construct two other frameworks based on our original proposed HMCNN-AC architecture, namely HMCNN and MCNN-AC, and compare their classification results with other baseline methods.

For the evaluation metrics of all methods above, we adopt overall accuracy (OA), average accuracy (AA), and κ coefficient [41] to measure the performance. OA is the overall accuracy for all classes and is defined as follow:

(15) $$\begin{array}{c} OA=\frac{\sum_{i}x_{\mathrm{test},\mathrm{correct}}^{\left(i\right)}}{N}, \end{array}$$

where $x_{\mathrm{test},\mathrm{correct}}^{\left(i\right)}$ is the *i*-th correctly classified test sample, *N* is the total number of test samples. AA represents the averaged accuracy of each class, and is defined as:

(16) $$\begin{array}{c} AA=\frac{1}{M}\sum_{i=1}^{M}\frac{\sum_{j=1}x_{i}^{\left(j\right)}}{N_{i}}, \end{array}$$

where *M* is the class number of each dataset, $N_{i}$ is the total test sample number of the *i*-th class, and $x_{i,\mathrm{correct}}^{\left(j\right)}$ is the *j*-th correctly classified test sample of class *i*. Kappa coefficient [41] is a statistical measurement of agreement degree, referred as κ. The higher of all our measurement metrics the better of the classification performance. The experiments are performed on a desktop PC equipped with an Intel Core 5 CPU and four GTX 1080 GPUs.

### 4.1. Dataset Description

**Figure 3:**
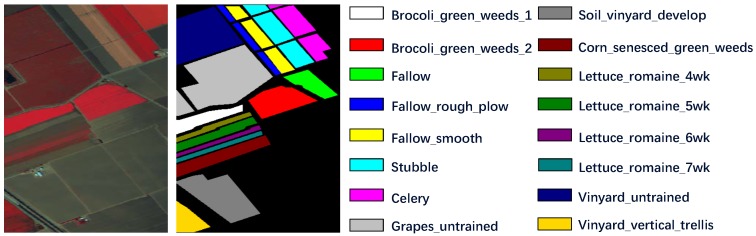
Salinas dataset description. The false-color image is generated from spectral band 52, 25, 10 and the groundtruth map together with the respective classes are displayed.

**Figure 4:**
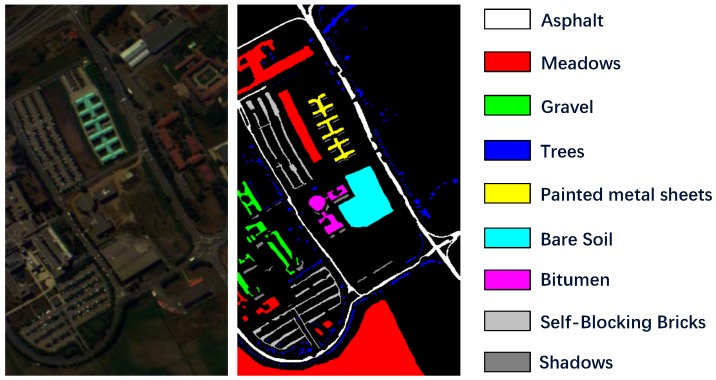
PaviaU dataset description. The false-color image is generated from spectral band 56, 28, 5 and the ground truth map together with the respective classes are displayed.

**Figure 5:**
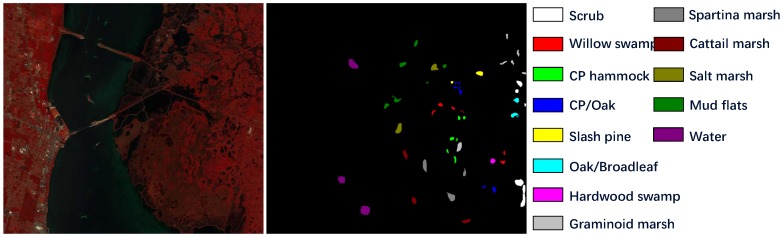
KSC dataset description. The false-color image is generated from spectral band 56, 28, 5 and the ground truth map together with the respective classes are displayed.

We choose Salinas, Pavia University (PaviaU) and Kenned Space Center (KSC) as our evaluation datasets and their corresponding false-color images and ground truth maps are shown as Figure 3, Figure 4 and Figure 5. Considering the dataset sizes, we randomly select 5% labeled samples of each class from Salinas and PaviaU as training sets and use the rest 95% samples as test sets. As for KSC, 10% samples of each class are chosen for training and the rest 90% samples for testing.

(1) Salinas Scene: This dataset was collected by AVIRIS sensor in 1992 which recorded the remote sensing images of Salinas Valley, CA, USA. The hyperspectral image cube contains 512 × 217 pixels in spatial dimension and 224 spectral bands. Owing to the noise’s influence, we discarded 20 noisy bands to generate an experimental dataset with only 204 spectral dimension. There are 16 different classes in this HSI, as shown in Figure 3. The training set and test set are presented in Table 2.
(2) Pavia University Scene: The Pavia University Scene was a hyperspectral image dataset captured by the reflective optics system imaging spectrometer (ROSIS) sensor in year 2001 over northern Italy. The original image consists of 610 × 340 pixels together with 103 spectral bands. Nine labeled material classes are available in this dataset, as shown in Figure 4. And the training data and test data are displayed in Table 3.
(3) Kenned Space Center: Our last dataset, Kenned Space Center (KSC), was acquired by the AVIRIS sensor in Florida on 23 March 1996. The spatial dimension of this hyperspectral image is 512 × 614 pixels and the original spectral dimension is 224 spectral bands. Due to the noisy bands, we only use 176 bands for method evaluation. The image set contains 13 labeled categories, which can be seen from the ground truth map in Figure 5. The training data and test data are described in Table 4.

### 4.2. Compared Methods

To demonstrate the effectiveness of our proposed HMCNN-AC, several classification approaches are adopted. These compared approaches are based on two kinds of groups: one is based on classical machine learning methods, which includes (RBF-)SVM and extended morphological profiles (EMP) [15]; the other is based on deep learning models, which includes SAE-PCA (joint spectral–spatial features) [20], CNN-MLR [22], LSTM [25], and MCNN [28]. Since the 3D-CNN method requires a long training time, here we exclude this method.

#### 4.2.1. Classical Machine Learning Methods

(a) The SVM method only utilizes spectral information of each pixel and we use the LibSVM [42] for the classification in our experiments. (b) For the EMP method, spatial features are exploited by the adoption of five opening and closing operations followed by morphological reconstruction on the first three principal components of HSI. We use the disk structure element to perform the morphological operations and the structure sizes range from 1 to 9. The generated features are then sent to a conventional RBF-SVM for classification.

#### 4.2.2. Deep Learning Based Methods

(a) For the implementation of SAE-PCA, first four PCs of each hyperspectral dataset are extracted and flattened within a certain neighborhood region, then they are concatenated with spectral features to obtain the joint spectral–spatial features for further processing. (b) For the CNN-MLR, the number of PCs is determined by reserving at least 99.9% information of the original hyperspectral dataset, then convolutional operations are applied to the reserved PCs on spatial dimension. (c) The LSTM method only exploits spectral information and regards a spectrum as sequential data to explore the spectral correlation within different spectral channels. (d) For MCNN, first three PCs are selected and three spatial scale patches for each PC band are generated and down-sampled to the equally spatial sizes for spatial feature extraction. The obtained spatial features are concatenated with spectral information for each scale’s classification and the final results are obtained by majority voting. Other parameter settings for SAE-PCA, CNN-MLR, LSTM and MCNN are set to the default values in [20,22,25,28], respectively.

### 4.3. Network Hyperparameters Discussion

**Figure 6:**
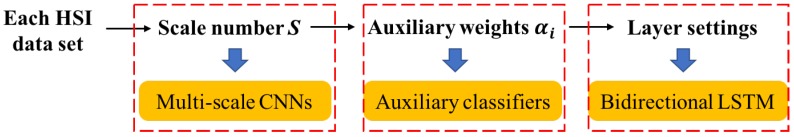
The flow chart of hyperparameter tuning process. For each HSI, we first fix auxiliary weights *α_i_* and layer settings in Bi-LSTM, and adjust the input scale number *S* to find the optimal one. Then we fix scale number *S* and layer settings in Bi-LSTM to find suitable auxiliary weights *α_i_*. The same procedure goes for determining suitable layer settings in Bi-LSTM. Finally, we obtain all the determined hyperparameters.

The hyperparameters in our proposed deep model could influence the classification performance. Except the weights in the networks can be automatically learned during the training process, several other related hyperparameters need to be discussed, such as input scale number *S*, auxiliary weights *α_i_* and layer settings in the bidirectional LSTM. In this section, we shall present the sensitivity analysis and investigate the effects of these hyperparameters, and the overall process is shown in Figure 6.

#### 4.3.1. Input Scale Number *S*

**Figure 7:**
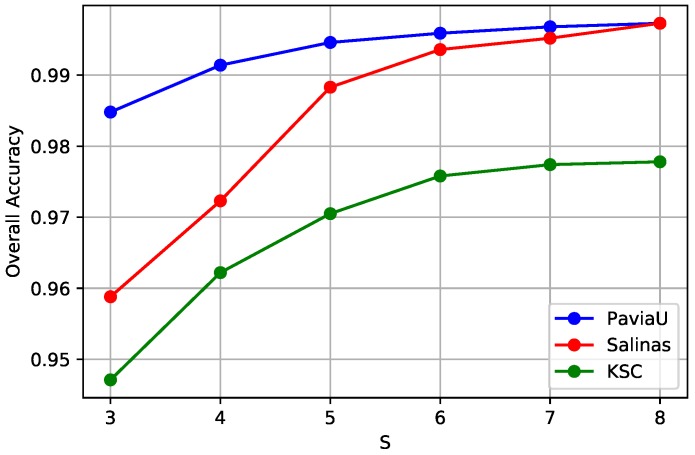
The curves of OA obtained by different input scale numbers *S* for three HSI datasets. The OAs first arise with the increase of the input scale number *S*, and then become saturated for all three HSIs. Thus, we choose the scale number *S* = 8 for all the HSIs for our proposed framework.

Objects in remote sensing images often appear with various shapes and scales, it is necessary to introduce multi-scale information into HSI classification. To quantitatively analyze the effects of different input scales on the final accuracy of our proposed method, scale number *S* from 3 to 8 with its corresponding largest input window *w* from 5 to 15 is investigated in this section. Figure 7 shows the OA value changes with scale number *S* on three HSI datasets. It can be seen that OAs first improve as the scale number *S* increases, but become saturated later on. On the one hand, classification with a small *S* fails to collect enough scale-dependent features, resulting in relatively lower accuracy. And a larger *S* allows us to consider more spatial scales, which helps to improve classification accuracy to some extent. On the other hand, further increasing *S* does not further improve performance since we already have enough spatial information. Moreover, it could incur an additional computational expense. Accordingly, we choose a spatial scale number of *S* = 8 with corresponding window size *w* = 15 for all three datasets.

#### 4.3.2. Auxiliary Weights *α_i_*

**Figure 8:**
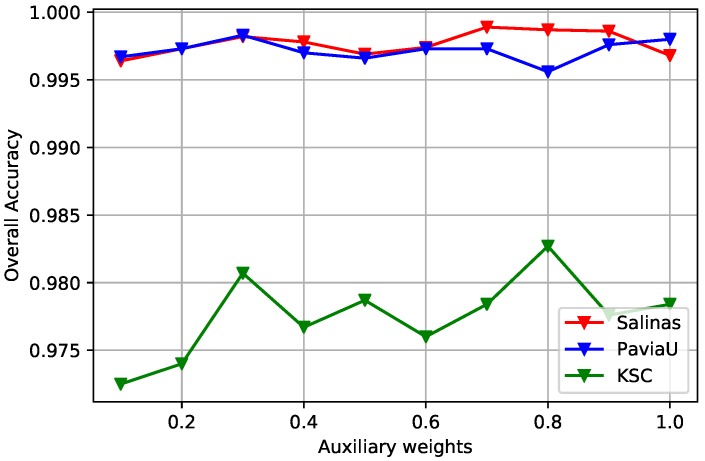
The curves of OA obtained by different multi-scale auxiliary weights for three HSI datasets. The auxiliary weights are set from 0.1 to 1, with an increment of 0.1. The optimal *α* is determined corresponding to the highest OA value for each HSI.

The auxiliary classifiers help to better train the multi-scale CNNs and obtain robust convolutional features. And the auxiliary weight $\alpha_{i}$ is an important parameter since it determines the percentage of each auxiliary loss $L_{aux}^{i}$ takes in the total loss *L*. In this study, we investigate the effects of auxiliary weights on the final classification results. To simplify the experiment, the auxiliary weights for each spatial scale are set to be equal $\alpha_{i}=\alpha$. Since the upper-scale patches always contain the lower-scale patches and we want to yield prediction of the central spectrum, equal weights assignment allows us to put more emphasis on the central spectra and reduce the unwanted noise. Specially, we set *α* from 0.1 to 1, with an increment of 0.1. The main loss weight is set to 1, as described in Equation (14). Figure 8 reports the classification results with different *α* values. It is clear that Salinas datasets with auxiliary weight *α* = 0.7, PaviaU dataset with *α* = 0.3, and KSC dataset with *α* = 0.8 achieved the best classification results.

#### 4.3.3. Layer Settings for Bidirectional LSTM

**Figure 9:**
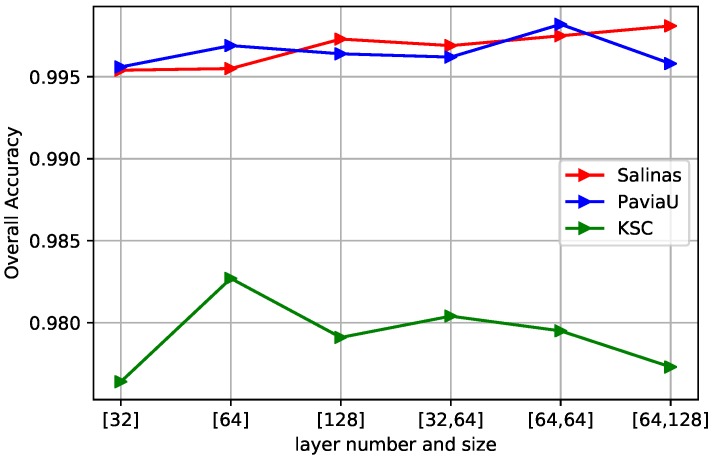
The curves of OA obtained by different layer numbers and hidden units in bidirectional LSTM. The optimal hyperparameter choice for Salinas is two-layer with 64 and 128 hidden units respectively, two-layer with 64 and 64 units for PaviaU, and one-layer with 64 hidden units for KSC due to smaller data size.

The bidirectional LSTM part in our proposed framework plays a crucial role in exploring the spatial–spectral dependency of multi-scale convolutional features and extracting a hierarchical representation for the final classification. In this section, the number of layers and the number of hidden units for the bidirectional LSTM are investigated from the set {[32], [64], [128], [32, 64], [64, 64], [64, 128]}, where the number of digits in each square bracket represents the layer number of the bidirectional LSTM, and the digits in square brackets indicate the unit numbers of their corresponding layers. Figure 9 shows the OA values with different layer settings. Accordingly, the optimal hyperparameters setting for Salinas is two-layer with 64 and 128 memory cells respectively. For Pavia University, two-layer with 64 memory cells respectively achieves best results. As for KCS datasets, one-layer with 64 memory cells yields good performance due to its smaller data size.

### 4.4. Classification Results

**Figure 10:**
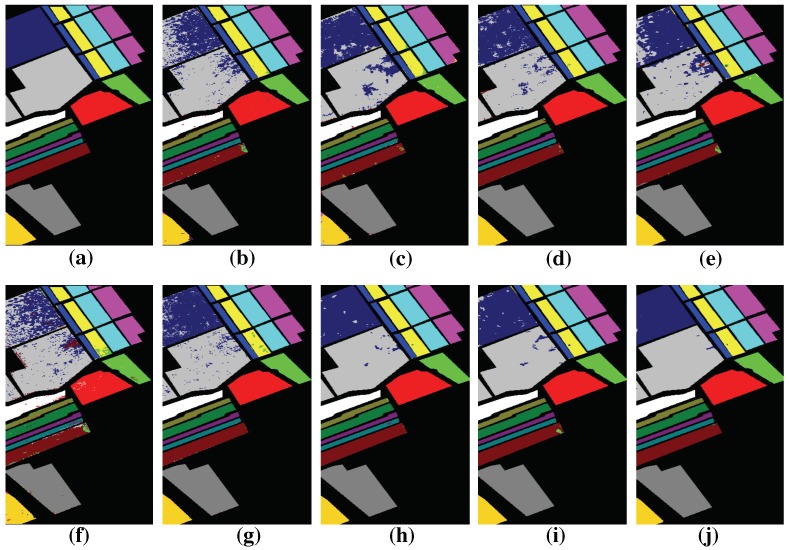
Groundtruth and classification maps with 5% training samples from Salinas dataset. (**a**) Groundtruth; (**b**) SVM; (**c**) EMP-SVM; (**d**) SAE-LR; (**e**) CNN-MLR; (**f**) LSTM; (**g**) MCNN; (**h**) HMCNN; (**i**) MCNN-AC; and (**j**) HMCNN-AC. The proposed HMCNN-AC yields the cleanest visualization maps with improved spatial consistency, which is most similar to the groundtruth map.

**Figure 11:**
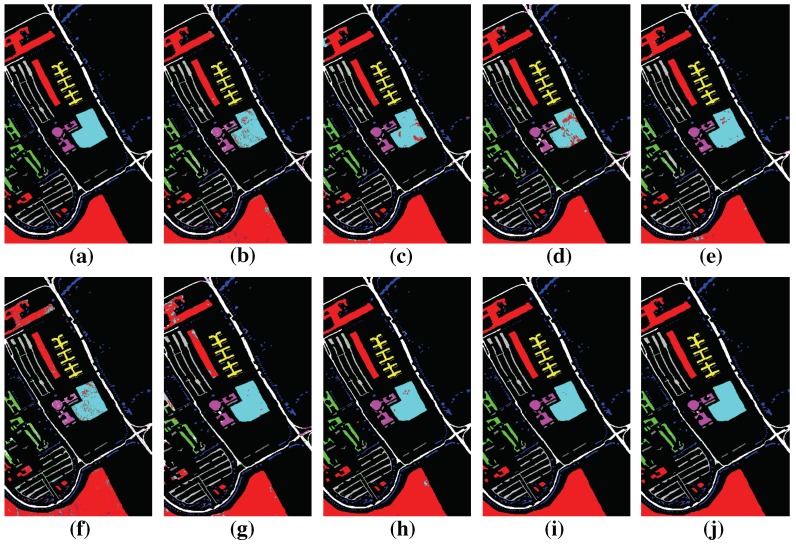
Groundtruth and Classification maps with 5% training samples from PaviaU dataset. (**a**) Groundtruth; (**b**) SVM; (**c**) EMP-SVM; (**d**) SAE-LR; (**e**) CNN-MLR; (**f**) LSTM; (**g**) MCNN; (**h**) HMCNN; (**i**) MCNN-AC; and (**j**) HMCNN-AC. The proposed HMCNN-AC yields the cleanest visualization maps with improved spatial consistency, which is most similar to the groundtruth map.

**Figure 12:**
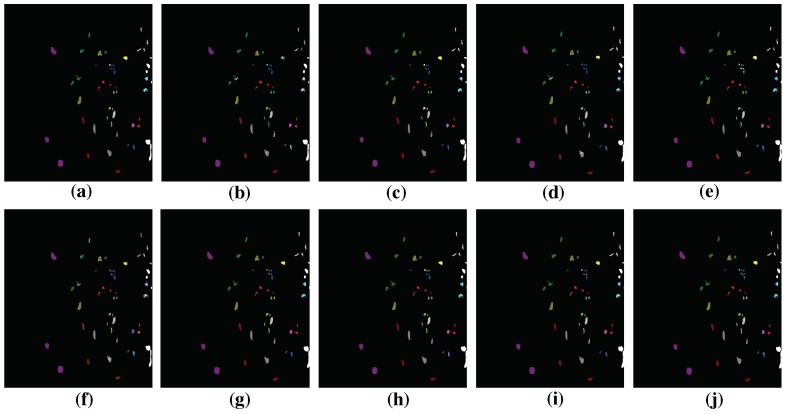
Groundtruth and Classification maps with 10% training samples from KSC dataset. (**a**) Ground truth; (**b**) SVM; (**c**) EMP-SVM; (**d**) SAE-LR; (**e**) CNN-MLR; (**f**) LSTM; (**g**) MCNN; (**h**) HMCNN; (**i**) MCNN-AC; and (**j**) HMCNN-AC. The proposed HMCNN-AC yields the cleanest visualization maps with improved spatial consistency, which is most similar to the groundtruth map.

In this section, we will report the classification results of the proposed HMCNN-AC along with other compared methods. The parameters are chosen based on our experimental results. To better train the whole network, batch normalization [43] and dropout technique [44] are adopted after every convolutional layer to avoid overfitting. We use RMSprop [45] as the optimization algorithm. The classification maps of different methods on three hyperspectral datasets are displayed in Figure 10, Figure 11 and Figure 12 and the quantitative assessments are shown in Table 5, Table 6 and Table 7. As can be seen, among all the methods, HMCNN-AC achieves the best classification results on all three datasets, with OA = 99.88% for Salinas with 5% training samples, OA = 99.83% for Pavia University with 5% training samples, and OA = 98.27% for Kenned Space Center with 10% training samples, and yields the cleanest visualization results much more similar to reference maps than others. Compared with other methods, SVM-RBF and LSTM obtain relatively poor performances and exhibit noisy estimations in classification maps, since they fail to consider spatial information. In contrast, the classification results of EMP-SVM, SAE, CNN-MLR and MCNN methods show much improvement and deliver smoother appearance in classification maps by combining spectral and spatial features, especially for MCNN method with multi-scale features.

The main reasons for the superior performance of our proposed HMCNN-AC lie in two aspects. On the one hand, multi-scale CNNs are adopted to capture the scale-dependent information considering objects with different shapes and sizes. With the help of auxiliary classifiers, the whole network could be better trained and robust convolutional features are acquired. On the other hand, to fully explore the spectral–spatial correlation of the obtained multi-scale features, a bidirectional LSTM is proposed to capture the dependency and extract a hierarchical representation for each pixel, instead of simply concatenating these multi-scale features together. In order to validate the effectiveness of the adopted auxiliary classifiers and the bidirectional LSTM, we construct two other frameworks, namely HMCNN and MCNN-AC, based on our original proposed HMCNN-AC architecture, and compare their classification performances on three HSIs with other baseline methods. The detailed analysis is shown as below.

### 4.5. Effective Analysis of Auxiliary Classifiers

**Figure 13:**
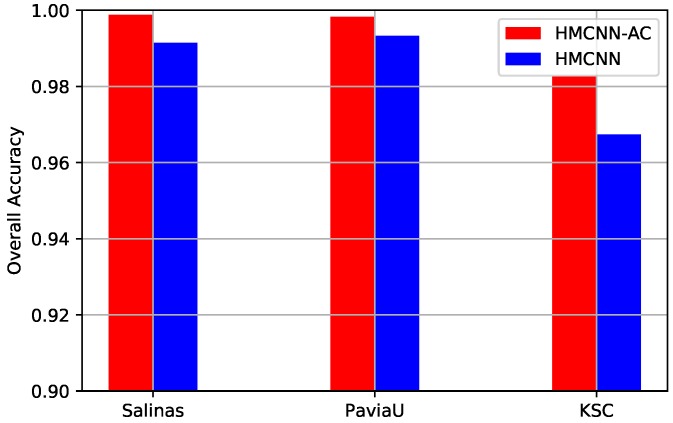
OAs comparison of HMCNN and HMCNN-AC on three datasets for effective analysis of auxiliary classifiers. For all three HSIs, OA value decreases for the constructed HMCNN framework, which confirms the effectiveness of the adopted auxiliary classifiers.

To further examine the effectiveness of the auxiliary classifiers, we construct another framework named HMCNN based on our original proposed HMCNN-AC architecture as the baseline. The network of HMCNN as well as other parameter settings is the same as our original proposed HMCNN-AC. The only difference between these two frameworks is whether adopting auxiliary classifiers. In that case, we could demonstrate the effectiveness of the auxiliary classifiers by comparing the classification performances of these two models on three HSI datasets. The quantitative assessments of HMCNN are also shown in Table 5, Table 6 and Table 7 with other baseline methods, and the visualization results can be seen in Figure 10, Figure 11 and Figure 12, subfigure (h). To clearly demonstrate the comparison results, we plot the OA values of the two proposed frameworks on three HSIs in Figure 13. The red bars represent the results of our original proposed HMCNN-AC with weighted auxiliary classifiers, and the blue ones indicate the newly constructed HMCNN without auxiliary classifiers. From the figure, it is clear to see a decline in OA for all three datasets with HMCNN architecture, which confirms the effectiveness of our auxiliary classifiers in improving HSI classification.

### 4.6. Effective Analysis of Bidirectional LSTM

**Figure 14:**
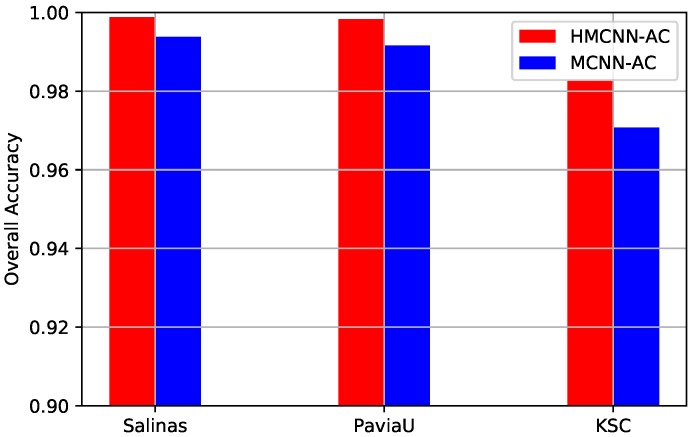
OAs comparison of the proposed MCNN-AC and HMCNN-AC on three datasets for effective analysis of bidirectional LSTM. For all three HSIs, OA value decreases for the constructed MCNN-AC framework, which validates the effectiveness of the adopted bidirectional LSTM.

To analyze the effectiveness of the adopted bidirectional LSTM in exploring the spectral–spatial correlation of the multi-scale features and learning hierarchical features, we replace the bidirectional LSTM part in our original proposed architecture with one simple concatenation layer as the baseline and keep other parameter settings the same. The constructed framework is named MCNN-AC and the concatenation layer concatenates the obtained multi-scale features into 1D vector for the final classification. The quantitative metrics and visualization maps of MCNN-AC on three HSIs are also compared with other methods in Table 5, Table 6 and Table 7 and Figure 10, Figure 11 and Figure 12, respectively. To better evaluate the effectiveness of adopting the bidirectional LSTM, we present the OA values of MCNN-AC and our original proposed HMCNN-AC on three datasets, as displayed in Figure 14. From the comparison, we can observe that the OAs of MCNN-AC decrease a little when replacing the original bidirectional LSTM with the concatenation layer, which validates the effectiveness of the bidirectional LSTM in spectral–spatial correlation exploration and hierarchical feature learning.

## 5. Conclusions

In this paper, a novel hierarchical multi-scale CNN with auxiliary classifiers (HMCNN-AC) is proposed for HSI classification. Unlike the conventional spectral–spatial classification methods where only one single input scale is considered for spatial feature integration, our proposed method could extract spectral–spatial features at various input scales simultaneously by adopting the multi-scale CNNs technique. To fully explore the spectral–spatial dependency of the obtained multi-scale features, a bidirectional LSTM is proposed to capture the correlation and extract a hierarchical representation for each hyperspectral pixel from a sequential perspective. In order to better train the whole network, weighted auxiliary classifiers are employed for the multi-scale CNNs and are optimized together with the main loss function. Experimental results on three public datasets demonstrate the superiority of our proposed method with the highest OA values and the cleanest visualization maps compared with other baseline methods.

Although our proposed HMCNN-AC model has achieved remarkable performance, some details are worth further investigation. For instance, the convolutional features from different spatial scales might exert different influence on the final classification and equal weights assignment would fail to reflect this difference in our current setup. Besides, the parameter settings in the multi-scale CNNs are also worth further discussion for better performance.

## Figures and Tables

**Table 1:** The network architectures of multi-scale CNNs. For multi-scale input patches ${\left\{P_{s}\right\}}_{s=1}^{S}$ with various input scales, the detailed structures of each sub-network including the numbers of convolution layers, kernel sizes and kernel numbers are specified.

Layer No.	*P*_1_ (1 × 1)	*P*_2_ (3 × 3)	*P*_3_ (5 × 5)	*P*_4_ (7 × 7)	*P*_5_ (9 × 9)	*P*_6_ (11 × 11)	*P*_7_ (13 × 13)	*P*_8_ (15 × 15)
1	1 × 1 × 32	1 × 1 × 32	3 × 3 × 32	3 × 3 × 32	3 × 3 × 32	3 × 3 × 32	3 × 3 × 32	3 × 3 × 32
2	1 × 1 × 32	3 × 3 × 32	3 × 3 × 32	3 × 3 × 32	3 × 3 × 32	3 × 3 × 32	3 × 3 × 32	5 × 5 × 32
3				3 × 3 × 64	5 × 5 × 64	3 × 3 × 64	5 × 5 × 32	5 × 5 × 64
4						5 × 5 × 64	5 × 5 × 64	5 × 5 × 64

**Table 2:** Groundtruth classes for Salinas scene and the corresponding number of training samples and testing samples for classification experiments.

Class Number	Class Name	Train	Test
1	Brocoli green weeds 1	100	1909
2	Brocoli green weeds 2	186	3540
3	Fallow	99	1877
4	Fallow rough plow	70	1324
5	Fallow smooth	134	2544
6	Stubble	198	3761
7	Celery	179	3400
8	Grapes untrained	563	10,708
9	Soil vinyard develop	310	5893
10	Corn senesced green weeds	164	3114
11	Lettuce romaine 4 wk	53	1015
12	Lettuce romaine 5 wk	96	1831
13	Lettuce romaine 6 wk	46	870
14	Lettuce romaine 7 wk	54	1016
15	Vinyard untrained	363	6905
16	Vinyard vertical trellis	90	1717
total		2705	51,424

**Table 3:** Groundtruth classes for PaviaU scene and the corresponding number of training samples and testing samples for classification experiments.

Class Number	Class Name	Train	Test
1	Asphalt	331	6300
2	Meadows	932	17,717
3	Gravel	105	1994
4	Trees	153	2911
5	Painted metal sheets	67	1278
6	Bare Soil	251	4778
7	Bitumen	66	1264
8	Self-Blocking Bricks	184	3498
9	Shadows	47	900
total		2136	40,640

**Table 4:** Groundtruth classes for KSC scene and the corresponding number of training samples and testing samples for classification experiments.

Class Number	Class Name	Train	Test
1	Scrub	76	685
2	Willow swamp	24	219
3	Cabbage palm hummock	26	230
4	Cabbage/oak hummock	25	227
5	Slash pine	16	145
6	Oak/broadleaf hummock	23	206
7	Hardwood swamp	11	94
8	Graminoid marsh	43	388
9	Spartina marsh	52	468
10	Cattail marsh	40	364
11	Salt marsh	42	377
12	Mud flats	50	453
13	Water	93	834
total		521	4690

**Table 5:** Classification accuracy (%) for Salinas dataset using 5% training samples via different classification algorithms. The three proposed frameworks HMCNN, MCNN-AC and HMCNN-AC outperform other baseline methods in results.

Class No.	SVM	EMP-SVM	SAE-PCA	CNN-MLR	LSTM	MCNN	HMCNN	MCNN-AC	HMCNN-AC
1	98.11	99.27	**100.00**	**100.00**	96.51	**100.00**	**100.00**	**100.00**	**100.00**
2	99.52	99.49	99.91	99.93	98.31	98.64	**100.00**	**100.00**	**100.00**
3	99.68	99.52	99.09	98.68	97.22	96.84	99.85	**100.00**	99.90
4	99.17	98.56	98.17	97.99	98.71	97.49	99.49	99.71	**100.00**
5	97.27	94.46	**100.00**	99.28	91.78	98.47	99.59	99.77	99.81
6	98.36	99.81	99.82	99.75	99.62	**100.00**	**100.00**	**100.00**	**100.00**
7	98.67	98.59	93.26	99.94	99.36	**100.00**	**100.00**	**100.00**	**100.00**
8	91.52	88.61	92.81	89.36	88.36	94.03	98.00	98.53	**99.86**
9	98.96	99.56	99.74	**100.00**	98.63	99.93	**100.00**	**100.00**	**100.00**
10	94.09	96.56	96.35	96.18	89.51	99.31	97.94	**99.78**	99.32
11	90.24	98.91	94.42	99.04	91.67	99.83	**100.00**	98.92	**100.00**
12	99.19	99.78	99.88	**100.00**	99.12	99.56	**100.00**	**100.00**	**100.00**
13	98.85	98.27	99.54	99.81	98.36	**100.00**	**100.00**	99.88	**100.00**
14	97.93	95.96	98.99	99.14	89.81	99.32	98.35	98.73	**100.00**
15	68.15	89.09	91.62	84.26	55.71	97.23	96.12	98.34	**99.74**
16	96.62	94.52	96.96	98.72	96.46	99.21	**100.00**	99.85	**100.00**
OA (%)	92.33	95.08	95.69	95.22	89.37	97.73	99.14	99.38	**99.88**
AA (%)	93.47	96.93	96.32	96.56	93.07	98.69	99.14	99.59	**99.91**
Kappa	0.9144	0.9453	0.9520	0.9470	0.8821	0.9742	0.9875	0.9932	**0.9987**

**Table 6:** Classification accuracy (%) for PaviaU dataset using 5% training samples via different classification algorithms. The three proposed frameworks HMCNN, MCNN-AC and HMCNN-AC outperform other baseline methods in results.

Class No.	SVM	EMP-SVM	SAE-PCA	CNN-MLR	LSTM	MCNN	HMCNN	MCNN-AC	HMCNN-AC
1	92.30	97.44	90.55	98.33	93.36	95.27	98.63	99.12	**99.68**
2	98.00	98.08	98.95	98.94	94.97	98.73	99.99	99.95	**100.00**
3	74.07	93.03	92.34	73.63	69.37	92.21	97.55	93.75	**98.72**
4	94.47	97.39	98.91	98.81	94.12	99.60	98.67	98.15	**99.73**
5	99.06	99.37	**100.00**	99.63	99.14	99.75	**100.00**	**100.00**	**100.00**
6	87.94	92.42	84.47	97.30	88.23	94.50	**100.00**	99.98	**100.00**
7	86.38	93.90	87.42	96.92	81.13	97.01	96.69	98.57	**99.40**
8	91.02	96.62	87.56	98.13	82.29	95.89	99.15	98.10	**99.67**
9	99.67	**100.00**	97.56	99.89	**100.00**	**100.00**	**100.00**	99.89	**100.00**
OA (%)	92.61	96.85	94.13	97.24	91.16	96.96	99.33	99.16	**99.83**
AA (%)	91.43	96.47	93.08	95.76	89.23	96.93	98.96	98.61	**99.69**
Kappa	0.9150	0.9582	0.9230	0.9647	0.8854	0.9625	0.9919	0.9893	**0.9976**

**Table 7:** Classification accuracy (%) for KSC dataset using 10% training samples via different classification algorithms. The three proposed frameworks HMCNN, MCNN-AC and HMCNN-AC outperform other baseline methods in results.

Class No.	SVM	EMP-SVM	SAE-PCA	CNN-MLR	LSTM	MCNN	HMCNN	MCNN-AC	HMCNN-AC
1	95.46	**100.00**	99.07	98.54	91.85	95.66	**100.00**	**100.00**	**100.00**
2	82.56	**100.00**	81.48	97.53	75.31	99.17	98.35	99.59	99.76
3	90.00	66.67	96.48	91.41	86.33	98.05	95.31	98.04	**99.22**
4	46.46	**99.56**	66.26	58.73	63.89	78.97	82.14	74.60	88.97
5	46.52	78.95	53.42	76.40	57.14	82.61	90.06	80.12	**93.17**
6	50.48	79.89	60.70	81.66	48.91	91.27	91.26	**95.63**	88.21
7	93.61	**100.00**	99.05	95.24	30.48	**100.00**	**100.00**	97.14	**100.00**
8	88.63	97.10	94.56	98.35	72.85	92.10	94.81	**98.76**	98.27
9	98.71	**100.00**	**100.00**	**100.00**	92.12	99.42	99.61	**100.00**	**100.00**
10	93.38	98.35	97.52	96.04	79.21	99.75	96.53	99.75	**99.85**
11	96.02	99.21	98.09	100.00	98.57	**100.00**	**100.00**	**100.00**	**100.00**
12	77.87	98.89	97.42	92.64	81.91	**99.60**	97.41	97.81	98.61
13	97.60	99.64	99.46	99.89	99.68	**100.00**	**100.00**	**100.00**	**100.00**
OA (%)	86.40	95.36	92.07	93.82	82.98	97.06	96.74	97.07	**98.27**
AA (%)	79.81	93.71	87.96	91.26	75.25	95.12	95.81	95.50	**97.39**
Kappa	0.8535	0.9528	0.9204	0.9380	0.8186	0.9620	0.9673	0.9707	**0.9758**
